# Development and Evaluation of Psychometric Properties of a Chinese Version Questionnaire for Measuring Emergency Nursing Interruptions

**DOI:** 10.1155/2024/8750135

**Published:** 2024-10-23

**Authors:** Tao Lin, Xianqiong Feng, Yongli Gao

**Affiliations:** ^1^Emergency Department of West China Hospital, Sichuan University/West China School of Nursing, Sichuan University, Chengdu, China; ^2^Institute of Disaster Medicine, Sichuan University, Chengdu, China; ^3^Nursing Key Laboratory of Sichuan Province, Chengdu, China; ^4^West China School of Nursing, Sichuan University/West China Hospital, Sichuan University, Chengdu, China

**Keywords:** emergency department, nursing interruptions, patient safety, questionnaire, reliability, validity

## Abstract

Nursing interruptions occur frequently and often have adverse outcomes, threatening patient safety. Emergency departments have a high incidence of nursing interruptions owing to the concentration of critically ill patients and frequent emergencies. Current research mainly focuses on large hospitals and uses observation and interview methods. Therefore, there is a need to develop tools for measuring emergency nursing interruptions. This study develops a survey questionnaire on emergency nursing interruptions. It tests its validity and reliability by building on the conceptual framework of emergency nursing interruption proposed in the literature. Specifically, we develop a test version of the emergency nursing interruption survey questionnaire using a Delphi expert inquiry and preinvestigation. We selected 1047 emergency nurses in 22 provinces and autonomous regions of China to participate in the survey by completing the questionnaire between June and July 2023 to evaluate the validity and reliability of the questionnaire. The final questionnaire comprised 26 items across 5 dimensions—sources, types, interrupted nursing activities, consequences of interrupted nursing activities, and management of nursing interruptions. The content validity indexes were 0.948 at scale level and 0.842–1.000 at item level. We used exploratory factor analysis (EFA) to extract five common factors with a cumulative variance contribution rate of 66.550%. The results of the confirmatory factor analysis (CFA) demonstrated a good model fit. Cronbach's *α* coefficient of the questionnaire was 0.912, split-half reliability was 0.846, and the retest reliability calculated using the intraclass correlation coefficient was 0.915. To ensure the structural validity of the scales, EFA and CFA were conducted using two different datasets. Thus, the questionnaire showed good validity and reliability and can be used to evaluate how nurses understand emergency nursing interruptions.

## 1. Introduction

Nursing interruptions refer to the external behaviors encountered by nursing staff during the provision of ethical care within a specified time role and environment, such as sudden interruptions or delays in current activities and distractions that divert their attention from the patient [1]. According to prior studies [2–4], hospitals experience at least one nursing interruption every 10 min, and over 90% of these interruptions can lead to adverse outcomes. This can reduce nurses' work efficiency; increase occupational burnout, clinical risks, and error rates; trigger adverse events; reduce medical quality; and threaten patient safety [2–4].

The emergency department is a high-risk area for nursing interruptions, and the work mode differs from that of other general departments. Its work is complex, busy, and multidisciplinary. Nursing work is challenging, and the risk of infection is high. Furthermore, there is a concentration of critically ill patients whose conditions are unstable. Emergencies co-occur, and staff must manage the urgency of information acquisition and the emotions of patients and their families. The overlapping of multiple tasks, the noisy environment, the change-of-pace environment, and the simultaneous rescue of multiple patients make emergency nursing work more difficult, resulting in frequent nursing interruptions in the emergency department, which may adversely affect patient health [5–8].

Currently, there are fragmented and localized studies on the current situation, core elements, adverse effects, and nursing interventions related to emergency nursing interruptions, both domestically and internationally [9, 10]. However, these studies have mainly focused on collecting data in large comprehensive hospitals through direct observation or interview methods [10–13], which limits the further deepening and promotion of interruption studies in hospitals at different levels and in different regions.

**Figure 1:**
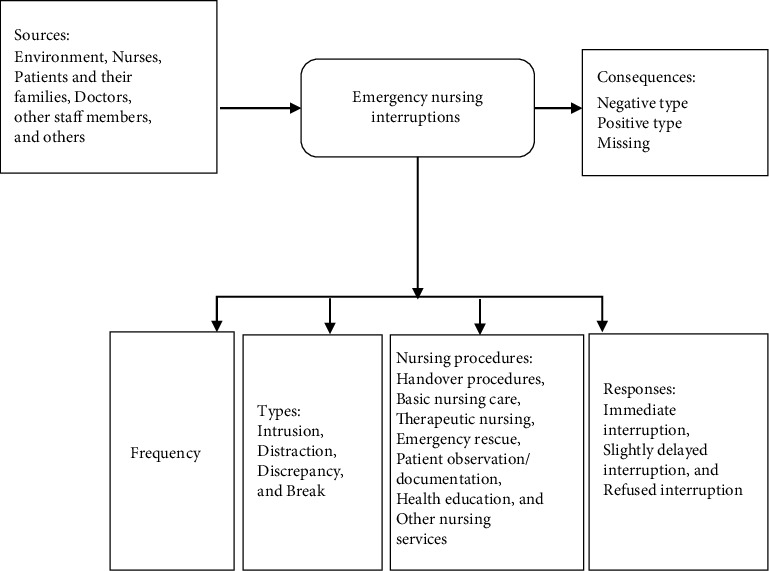
Theoretical framework. Note: (1) Breaks are planned or spontaneous recesses taken during work from a task that interrupts the flow and continuity of the task. (2) Negative type refers to interruption events that temporarily interrupt the nursing process and produces one of the following results: (i) extends the completion time of a certain job/operation; (ii) increases the workload; (iii) affects the quality of nursing work; (iv) causes adverse nursing events. (3) Positive type implies that temporary interruption has a positive impact on nursing procedures and results and that timely termination can prevent or avoid adverse consequences—such as when medical staff find potential medical errors and take the initiative to interrupt, correct, and remind each other. (4) Missing consequences: the primary reason for this is that the observers failed to monitor the entire interruption process as well as the interruption results. This occurred when two interruptions overlapped, which hindered the observer's ability to capture the results of the previous interruption.

To expand this research stream, we referenced the relevant theories of interruption as follows: (1) Interruption process theory [14] emphasizes that the interruption process is behavioral, starting with the original task, prompting the appearance of the interruption task, inputting the interruption task, ending the interruption task, and completing the original task. This theory can guide research teams to understand the process of interruption generation. (2) The theory of the impact of interruption on prospective memory [15] examines how nurses often face multiple tasks and frequent interruptions. The phenomenon of forgetting to perform specific tasks at a preordained time is called *prospective memory retrieval failure*. The occurrence of interruptions significantly affects the expression of this prospective memory. (3) The interruption management stage model [16] provides operator interrupt management and describes the negative impact of interruptions and people's management methods. We proposed a conceptual framework for emergency nursing interruptions based on the above theories [7]. This framework includes several interconnected parts, including inputs (mainly the sources of interruption), attributes—including interruption frequency, types, numbering procedures, and responses—and outputs (interruption consequences) (Figure 1). We also developed a multidimensional emergency nursing interruption questionnaire based on the observational and qualitative research results of our research group [6, 7, 17], which included the sources, types, interrupted nursing activities, consequences of interrupted nursing activities, and management of nursing interruptions. The reliability and validity of the questionnaire were tested among emergency nurses in China to provide new survey tools for emergency nurses to research nursing interruptions.

## 2. Research Methods

### 2.1. Preparation of the Initial Draft Questionnaire

#### 2.1.1. Theoretical Framework

This study is based on the theoretical framework of emergency nursing interruptions, extant observational, and qualitative studies [6, 7] that have identified several core elements of emergency nursing interruptions and the characteristics of emergency nursing work in China. The preliminary questionnaire included five dimensions—source of interruption, type of interruption, interrupted nursing activities, consequences of interrupted nursing activities, and management of nursing interruptions.

#### 2.1.2. Establishment of the Initial Item Pool

We searched the China National Knowledge Infrastructure, China Biomedical Literature Database, the Wanfang Database, and the VIP Database for Chinese search terms such as “interruption,” “work interruption,” “nursing interruptions,” and “emergency nursing interruptions.” Furthermore, we searched the Web of Science and PubMed databases using the English search terms “interrupt,” “nursing interruptions,” and “emergency.” The search period was from the database establishment to August 2022. We referred to the theoretical framework of emergency nursing interruptions, combined with the specific situation of China and the characteristics of emergency work. We organized a meeting of nurses in the emergency department of the investigator's hospital to discuss and brainstorm the articles extracted from the literature. We then summarized, resummarized, and deleted questions to form the initial version of the questionnaire, which comprised 26 items.

### 2.2. Consultation With Experts

#### 2.2.1. Selection of Experts

Using a purposive sampling method, we selected 18 experts to participate in a Delphi inquiry. We included experts who: (1) were engaged in nursing management, emergency medical/nursing, nursing education, nursing research, and other related fields; (2) had a bachelor's degree or above; (3) had intermediate and above professional titles; (4) had more than 10 years of work experience; (5) were familiar with emergency nursing work or questionnaire preparation; and (6) were willing to provide informed consent and participate voluntarily. We selected six content validity evaluation experts with over 15 years of work experience, senior professional titles, and mastery of questionnaire preparation methods.

We informed the invited experts of the purpose and significance of this study and obtained their consent in advance of the consultation. We sent the inquiry form to the experts by email. This included the explanation letter, general information, the rationale for the questionnaire, and the importance and relevance scores of the articles. Experts were invited to provide their opinions on the professional content, expression, and formatting of the questionnaire. The article pool of the preliminary questionnaire was added, and items were deleted and modified according to the expert opinions to ensure the representativeness and clinical applicability of the questionnaire.

#### 2.2.2. Development of Inquiry Forms and Selection Methods for Entries

The form developed in this study through expert consultation included the following parts: (1) terminology, introduction of the research background, purpose, and content of this study; (2) basic information such as gender, age, job title, and field of study; and (3) expert evaluation of the relevance, importance, and feasibility of the survey questionnaire items about the occurrence of emergency nursing interruptions. The selection criteria for expert inquiry items were mean importance score > 3.50 points, coefficient of variation < 0.25, and total score ratio > 20% [18]. After objective evaluations, the research team—including master's student supervisors, clinical nursing experts, nursing administrators, nursing graduate students, and clinical nurses—conducted group discussions based on expert opinions and applied item selection criteria to make appropriate choices and modifications of the questionnaire items, resulting in a presurvey version.

### 2.3. Preinvestigation

We used purposive sampling to select 30 members from the nursing staff of the emergency department of a tertiary Grade A comprehensive hospital in Chengdu, Sichuan Province, China, in May 2023. We used a test version of the questionnaire as a preliminary survey to test whether the expressions of each item were easy to understand and unambiguous. Inclusion criteria for survey participants were those: (1) aged ≥ 18 years old; (2) possessing a nursing qualification certificate and being on duty; (3) with 1 year or more of emergency work experience; and (4) who agreed to participate in this study voluntarily. Individuals who were continuing education or regular training and rotation and internship nurses were excluded from the study. We distributed 30 questionnaires (in Chinese languages) using the QuestionStar platform and collected 30 valid questionnaires for a 100% effective response rate. Based on the preliminary survey results, we made minor language modifications to the items and developed an official test version of the emergency nursing interruption survey questionnaire.

### 2.4. Formal Questionnaire Testing

#### 2.4.1. Survey Participants

From June to July 2023, we used the Emergency Nursing Professional Committee of the Chinese Nursing Society to contact emergency committees in various provinces, autonomous regions, and municipalities across the country. The emergency committees of each nursing society contacted the nursing department heads of the hospitals at all levels in their respective regions. They informed the department heads of the purpose and significance of this study. After obtaining consent from emergency nurses at the institute's hospital, we sent electronic questionnaire links to the hospitals. The hospitals arranged for lower-level emergency department nurses to participate. The inclusion and exclusion criteria for survey participants were the same as those for the preliminary survey.

#### 2.4.2. Survey Tools

We distributed 1208 questionnaires and collected 1047 valid responses. Therefore, the valid questionnaire response (based on the number of questions responded to in a questionnaire) rate was 86.67%. The number of valid questionnaires collected in June and July 2023 was 314 and 733, respectively. The 314 cases surveyed in June 2023 were used for item analysis, reliability testing, and exploratory factor analysis (EFA) of the questionnaire. The 733 cases surveyed in July 2023 were used for the confirmatory factor analysis (CFA). The sample size for this study met the requirements for CFA, having 5–10 times the number of questionnaire items [19] and over 200 cases [20] (accounting for a sample stripping rate of 10%–20%).

### 2.5. Statistical Methods

We used Excel 2016, SPSS 26.0, and AMOS 26.0 software for data entry and statistical analyses. We described the measurement data using means and standard deviations (SDs), while count data were expressed as frequencies and percentages. The screening and evaluation of the questionnaire items were conducted using the item analysis method; validity was evaluated using EFA, CFA, and the content validity index. Reliability was assessed using Cronbach's *α* coefficient, split-half reliability, and retest reliability. The retest reliability was calculated using the intraclass correlation coefficient (ICC). Statistical significance was set at *p* < 0.05.

### 2.6. Validity and Reliability Testing

#### 2.6.1. Item Analysis

We used item analysis to test the discriminability of scale entries [21]. The filtering criteria for the entries were as follows: (1) Correlation coefficient method: We deleted items with a correlation coefficient < 0.4 with the total score of the scale; (2) critical ratio method: We sorted the data by total score, compared the differences in scores between the low group (top 27%) and the high group (bottom 27%), and deleted items which lacked statistically significant differences (*p* > 0.05); (3) internal consistency coefficient method: We deleted the items for which the Cronbach's *α* coefficient of the scale increased significantly after removing them.

#### 2.6.2. Validity Testing

Validity refers to the degree to which a research tool can accurately reflect the expected research concept [22]. Content validity is divided into item-level CVI (I-CVI) and scale-level CVI (S-CVI) at the questionnaire level. In this study, six experts were invited to rate the correlation between each item of the questionnaire and its relevant dimensions using a four-level scoring method, with one point indicating “uncorrelated,” two points “weakly correlated,” three points “correlated,” and four points “highly correlated.” It is generally believed that I-CVI > 0.75 and S-CVI ≥ 0.90 indicate good content validity [22]. We used EFA to evaluate structural validity. EFA uses principal axis factorization to extract common factors without limiting the number of factors. The criteria are as follows: extracting factors with eigenvalues > 1, cumulative variance contribution rate of factors > 60%, number of items under common factors ≥ 3, and loading value of each item on only one factor ≥ 0.4 [23]. We used CFA to evaluate convergent validity. CFA uses the maximum likelihood method to calculate chi-squared values/degrees of freedom (*χ*^2^/df), root mean square error of approximation (RMSEA), incremental fit index (IFI), Tucker–Lewis index (TLI), comparative fit index (CFI), and parsimony-adjusted goodness-of-fit index (PGFI). The simplicity-adjusted normalized fit index (PNFI) evaluates model fit.

#### 2.6.3. Reliability Testing

Reliability refers to the degree of consistency or accuracy of the results obtained using a research tool [22]. We used Cronbach's *α* coefficient (the most used reliability coefficient), split-half reliability, and test–retest reliability to assess the reliability of the developed questionnaire.

### 2.7. Ethical Considerations

This study was approved by the ethics committee of the study hospital (West China Hospital of Sichuan University [No. 966] in 2021). Informed consent was obtained from all participants before their participation in the study. This study was conducted by the Declaration of Helsinki.

## 3. Results

### 3.1. Expert Inquiry Results

From February to March 2023, we selected 18 experts (two men and 16 women) from Beijing, Shanghai, Sichuan, Shandong, Shaanxi, Jilin, Henan provinces, Chongqing Municipality, and the Guangxi Zhuang Autonomous Region. Their ages ranged from 36 to 59 (46.43 ± 7.26) years and included six undergraduates, nine master's, and three doctoral experts. Work experience ranged from 11 to 41 (22.08 ± 9.14) years. There were four intermediate professionals, six deputy senior professionals, and eight senior professionals. Two were emergency medical personnel, six were emergency nursing personnel, two were involved in nursing research, and eight were in nursing management. Two rounds of expert inquiry were conducted, with expert enthusiasm coefficients of 87.42% and 91.35%, respectively. The expert authority coefficients were 0.846 and 0.862, respectively. The Kendall harmony coefficients were 0.174 and 0.181, respectively (*p* < 0.001).

In the first round of expert inquiry, the average score of the importance of the items was 3.85–4.90, with a coefficient of variation of 0.05–0.41, and a total score ratio of 37.64%–96.68%. The coefficient of variation is typically calculated by dividing the SD by the mean. This indicates the extent of dispersion in the data distribution. The coefficient of variation of 0.05–0.41, as referenced in the results indicates that the degree of variation in expert ratings varies considerably across items. This suggests that while some items exhibit a high degree of consistency, others demonstrate a lower degree of consistency. The total score ratio may be understood as the importance score of the item as a percentage of the total possible score. If the scale ranges from 1 to 5, a score ratio of 37.64%–96.68% indicates that certain items have near-perfect importance scores, while other items enjoy lower scores uniformly. The overall score ratio is typically calculated by dividing the sum of the item scores by the highest possible score. A total of 18 experts provided opinions, and after discussion, 15 items were modified or merged, one item was deleted, three new items were added, and ultimately, 28 items in five dimensions were retained. We deleted the item “Do you think the outcome of emergency nursing interruptions is mostly negative, with the focus on extending the completion time of a certain task or operation?” We added the following items: “I believe that the most common sources of nursing interruption are from various types of students (e.g., continuing education, regular training, rotation, internships, and specialized nurses),” “I believe that the most easily interrupted nursing function is emergency triage,” and “I believe that the outcome of emergency nursing interruptions has no impact on nursing work.”

In the second round of expert inquiry, the average score for the importance of the items was 4.42–4.96, with a coefficient of variation of 0.05–0.21 and a total score ratio of 52.94%–94.12%. Six experts (the remaining 12 experts had no revision opinion) proposed modifications. After discussion, six items were modified; however, no new items were added. Finally, five dimensions and 28 items were retained.

### 3.2. Participant Characteristics

We officially surveyed 1047 nurses; the specific demographic data are shown in Table 1.

### 3.3. Item Analysis Results

The item analysis results showed that the correlation coefficient between each item and the total score was 0.604–0.850 (*p* < 0.05). The difference between the scores of each item in the high and low groups was statistically significant (*p* < 0.05), and the discrimination of each item was good. Overall, Cronbach's *α* of the initial questionnaire draft was 0.964. There was no entry in which Cronbach's *α* coefficient was significantly increased after deleting this entry. We retained all items with significantly improved coefficients.

### 3.4. Content Validity

According to the expert evaluation results, the S-CVI of the entire questionnaire was 0.948, and the I-CVI of each item was 0.842–1.000.

### 3.5. EFA Results

A preliminary EFA was conducted on 28 items, and the results showed a Kaiser–Meyer–Olkin (KMO) value of 0.914 and a Bartlett's sphericity test value of *χ*^2^ = 5230.905 (*p* < 0.001), indicating suitability for factor analysis. After multiple EFA, combined with the factor loading situation of the items and the discussion of the research group on the content of the items, two items were deleted. Two items were excluded from the analysis because they exhibited low loading on a single factor or significant cross-loading across multiple factors, rendering them unattributable to a single factor. This may have contributed to an overall reduction in the structural validity of the scale. The two items deleted were as follows: “I believe that the most common sources of nursing work interruption are other people outside the above-mentioned personnel” and “I believe that bedside teaching is the most common source of interruption in nursing work.” The total scale's Cronbach's *α* coefficient was 0.906; however, when items SI2 (0.902) and INA5 (0.904) were removed, the Cronbach's *α* coefficient increased. EFA was conducted on the 26 remaining items. The results showed a KMO value of 0.917 (greater than 0.7) and Bartlett's sphericity test value *χ*^2^ = 5028.274 (*p* < 0.001), given a significance level of 5%, indicating suitability for factor analysis.

Table 2 shows the factor analysis results for the 26 items of the emergency nursing interruption questionnaire. According to the principle of eigenvalues greater than one, five main factors were extracted from the scale. The cumulative variance contribution rate of these five main factors reached 66.55%, indicating that the model was able to account for the majority of the data variance in a reasonable manner. However, it should be noted that some information was not captured, and the factor analysis results were reliable. In the table, SI1–SI7 have a larger load on Factor 2 and can be called the source factor of the interruption; TI1–TI4 have a larger load on Factor 4 and can be called the type factor of interruption; INA1–INA9 have a significant load on Factor 1 and can be called the interrupted nursing activity factor; OINA1–OINA5 have a significant load on Factor 3 and can be called the outcome factor for nursing activity interruption; and HNIE1–HNIE3 have a significant load on Factor 5 and can be called the processing factor for nursing interruptions. All factor loadings were greater than 0.5, and there was no severe cross-loading for any item. Each measurement item was clustered under a corresponding factor, indicating that the questionnaire had good structural validity.

### 3.6. CFA Results

As shown in Table 3, the *χ*^2^/df value before correction was 5.045, which is greater than 5. The value of TLI was 0.896, which was less than 0.9. Overall, the adaptability of the prerevision emergency nursing interruption questionnaire was not ideal. We corrected the model by pulling the correlations between residual terms with higher modification indices values. The corrected *χ*^2^/df value was 4.661, which is below 5. The RMSEA value was 0.071, which was less than 0.08. The values of IFI, TLI, and CFI were 0.917, 0.906, and 0.916, respectively, which were all greater than 0.9. The PGFI and PNFI values were 0.709 and 0.794, respectively, both greater than 0.5. Overall, the indicators of the revised emergency nursing interruption questionnaire were well-adapted.

**Figure 2:**
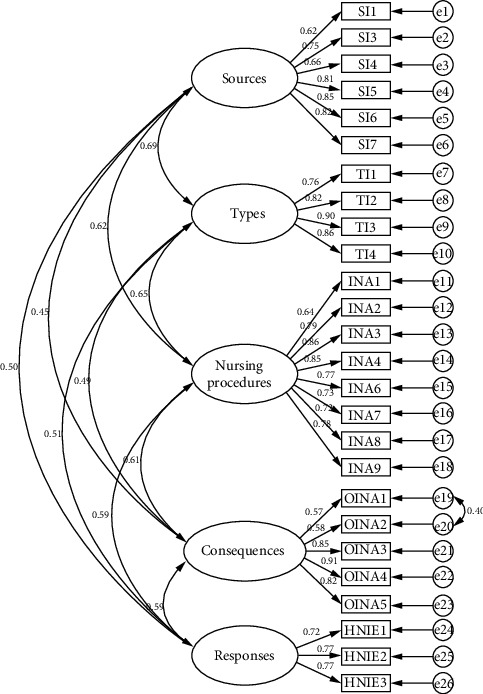
Standardized path coefficient diagram of the confirmatory factor analysis model for the survey questionnaire on emergency nursing interruptions.

The degree of fit of the model is shown in Table 4, and the CFA model is shown in Figure 2.

Table 4 shows the convergent validity table of the questionnaire, with three criteria for evaluating convergent validity: (1) All standardized factor weights must be greater than 0.5; (2) the composition reliability (CR) should be greater than 0.6; and (3) the average variance extraction (AVE) should be greater than 0.5. As shown in the table, the standardized load values of each item in the questionnaire were all greater than 0.5; CR values of the source, type, nursing activity being interrupted, outcome of nursing activity being interrupted, and management of nursing interruptions were all greater than 0.6. The AVE values were all greater than 0.5, indicating that the convergent validity of the questionnaire met the standards.

### 3.7. Reliability Analysis Results

The total Cronbach's *α* coefficient of the questionnaire was 0.912, and those for each dimension were between 0.876 and 0.950. The total split-half reliability of the questionnaire was 0.846, and the split-half reliability of each dimension was 0.782–0.875. We selected 20 survey participants for retesting within a 2-week interval. The 20 nurses were selected from the emergency department of a general Grade III hospital in Mianyang City, Sichuan Province, China. The retest reliability was calculated using the ICC—a value of 0.915 was obtained, and the retest reliability for each dimension was 0.886–0.936 (Appendix 1 and 2).

## 4. Discussion

### 4.1. Innovation and Significance of the Questionnaire

Nursing interruptions occur frequently in clinical work and often lead to negative outcomes. The effective management of emergency nursing interruptions is conducive to improving the quality of nursing and ensuring the safety of emergency patients. Currently, a specific tool to assess the interruptions in emergency care is lacking. To address this gap, this study developed and validated an emergency nursing interruption questionnaire to assess the interruptions in emergency care.

Emergency nursing interruptions not only increase the workload of nurses and the number of contradictions between doctors and patients but also reduce the professional identity of emergency nurses [7]. Emergency nurses play an important role in emergency medical activities, which is closely related to the treatment effect and safety of patients [5, 24, 25]. The significance of reducing the occurrence of emergency nursing interruptions is to improve the quality of nursing and help to ensure the safety of emergency patients and medical workers. In this regard, the results of the emergency nursing interruption questionnaire will be used to manage such interruptions. After the precise evaluation of the developed questionnaire, targeted interventions for emergency nursing interruptions can be further developed and implemented [24, 25]. To improve the quality of nursing and ensure the safety of patients, emergency managers should deeply explore the causes of nursing interruptions, analyze them, and develop more suitable strategies to manage adverse nursing events.

### 4.2. Scientific Rigor of the Questionnaire

This study was based on the theoretical framework of emergency nursing interruptions constructed in our preliminary research group. Based on the literature review and group discussions, we constructed an initial item pool comprising five dimensions—source of interruption, type of interruption, nursing activities that were interrupted, consequences of nursing activities that were interrupted, and management of nursing interruptions. We invited 18 experts from nine provinces for two rounds of expert inquiry to screen and revise the questionnaire items to determine the item pool. The positive coefficient of experts in the two rounds of expert inquiry was > 80.00%, and the authority coefficient of experts was > 0.800. The Kendall harmony coefficient significance test was significant (*p* < 0.001), indicating good consistency of expert opinions and high recognition of the questionnaire items [26]. We combined item analysis for item selection to improve the questionnaire discrimination and make the questionnaire more concise and scientific [27]. The S-CVI of the entire questionnaire was 0.948, and the I-CVI of each item was 0.842–1.000. According to the content validity evaluation criteria, the questionnaire's content validity was good [28]. The cumulative variance contribution rate of the five common factors extracted from EFA was 66.55% (> 60%), and the load values of each item on the corresponding factors were all > 0.5, indicating that the questionnaire had good structural validity. The results of CFA showed that the modified fitting indicators of each model met the fitting standards, indicating that the model was well adapted, and the questionnaire factor structure was relatively stable [20]. The total Cronbach's *α* coefficient of the questionnaire, split-half reliability, and retest reliability were all > 0.7, indicating that the questionnaire has good reliability [6]. The validity and reliability testing results indicated that the questionnaire has high scientific validity.

### 4.3. Clinical Practicability of the Questionnaire

The questionnaire contained a moderate number of items and was easy to understand and use. Generally, self-assessment can be completed within 10 minutes, making it convenient for clinical nurses. The five dimensions of this questionnaire comprehensively reflect the content of emergency nursing interruptions and measure their levels. This provides a preliminary solution for the lack of quantitative evaluation tools and the difficulty of conducting large-scale surveys. Based on the evaluation tools, researchers can conduct a series of studies on the current situation, influencing factors, mechanisms of action, intervention strategies, and evaluation of the intervention effects of emergency nursing interruptions to further explore management strategies for emergency nursing interruptions. This study is expected to provide a reference for researchers, educators, and managers when conducting related work.

Nursing managers should guide hospitals' nursing safety teams to provide safety training to emergency departments (high-risk departments for nursing interruptions) and their clinical nurses, establish awareness of nursing interruptions, and strengthen clinical safety behaviors [3, 24, 25, 29]. Simultaneously, precise intervention and strengthened training should be conducted to address the frequent occurrence of nursing interruptions in clinical practice, implement patient safety goals, create a “noninterruption event” ward, promote the construction of department safety culture, and improve the quality of nursing work [29, 30]. By conducting systematic analysis, education, planning, research, and quality improvement, we can avoid the occurrence of nursing interruptions with adverse outcomes [24, 25, 31].

### 4.4. Limitations

This study has certain limitations. First, although the sample size met the statistical requirements, the number of surveyed participants was limited. It is recommended that this scale be used in emergency departments in different regions to verify its universality. Second, owing to the different national conditions and emergency settings in different countries, further adjustments will be required to extend the results of this study to other countries.

## 5. Conclusions

This study constructed an emergency nursing interruption survey based on a standardized questionnaire development process. The questionnaire comprised five dimensions and 26 items. The questionnaire demonstrated good validity, reliability, scientificity, and practicality; thus, it enables the multidimensional evaluation of emergency nursing interruptions. By meeting the needs of frontline clinical nurses in China, we can improve the management of interruption events, thereby reducing the adverse effects of interruption events and ensuring patient safety.

## 6. Implications for Nursing Management

Emergency nursing managers should learn more advanced management models and scientific management tools to prevent the occurrence of emergency nursing interruptions with adverse outcomes, manage the root causes of adverse events, improve the coping ability of nursing staff when the interruption occurs, and improve the outcome of nursing interruptions. These measures will improve nursing quality and ensure the safety of patients and medical workers.

## Figures and Tables

**Table 1:** General information of the respondents (*n* = 1047).

	Number of cases (percentage)
Sex	
Male	185 (17.7)
Female	862 (82.3)
Age (years)	
18–25	172 (16.4)
26–30	275 (26.3)
31–40	451 (43.1)
41–50	115 (10.9)
>50	34 (3.3)
Marital status	
Unmarried	338 (32.3)
Married	680 (64.9)
Divorced or other	29 (2.8)
Education background	
Technical secondary school	7 (0.8)
Junior college	209 (19.9)
Undergraduate	806 (76.9)
Master's or above	25 (2.4)
Professional title	
Nurse	170 (16.2)
Nurse practitioner	419 (40.1)
Head nurse	386 (36.9)
Associate director nurse	64 (6.1)
Director nurse	8 (0.7)
Experience in emergency nursing (years)	
1–5	417 (39.8)
6–10	279 (26.6)
11–15	208 (19.9)
16–20	68 (6.5)
>20	75 (7.2)

**Table 2:** Exploratory factor analysis results of the questionnaire on emergency nursing interruptions (*n* = 314).

	Component				
	1	2	3	4	5
SI1	0.087	**0.643**	0.113	0.265	0.380
SI3	0.243	**0.677**	0.092	0.143	−0.001
SI4	0.246	**0.741**	0.169	0.212	0.060
SI5	0.203	**0.730**	0.217	0.210	0.125
SI6	0.279	**0.658**	0.203	0.153	0.190
SI7	0.140	**0.809**	0.135	0.151	0.092
TI1	0.366	0.213	0.046	**0.707**	0.088
TI2	0.276	0.203	0.095	**0.804**	0.100
TI3	0.232	0.251	0.143	**0.788**	0.079
TI4	0.168	0.267	0.145	**0.784**	0.130
INA1	**0.695**	−0.014	0.107	0.086	−0.034
INA2	**0.703**	0.322	0.053	0.122	0.161
INA3	**0.647**	0.345	0.111	0.267	0.282
INA4	**0.671**	0.295	0.042	0.305	0.174
INA6	**0.726**	0.192	0.160	0.238	0.021
INA7	**0.730**	0.142	0.284	0.071	−0.006
INA8	**0.609**	0.290	0.276	0.213	0.114
INA9	**0.735**	0.197	0.009	0.258	0.258
OINA1	0.108	0.221	**0.742**	−0.038	0.005
OINA2	0.043	0.112	**0.726**	−0.068	0.193
OINA3	0.238	0.139	**0.724**	0.235	0.172
OINA4	0.220	0.108	**0.782**	0.193	0.179
OINA5	0.123	0.161	**0.715**	0.271	0.255
HNIE1	0.092	0.183	0.232	0.095	**0.710**
HNIE2	0.154	0.136	0.124	0.179	**0.784**
HNIE3	0.103	0.076	0.241	0.013	**0.786**
Characteristic value	10.276	2.435	1.772	1.455	1.365
Variance percentage	39.523	9.367	6.815	5.596	5.249
Accumulated variance percentage	39.523	48.890	55.705	61.301	66.550

*Note:* Bold is the high factor load of each column, in order to determine which factor each item belongs to.

Abbreviations: HNIE, handling of nursing interruption events; INA, interrupted nursing activities; OINA, the outcome of interrupted nursing activities; SI, sources of interruption; TI, types of interruption.

**Table 3:** Results of fitting indicators for the questionnaire model of the emergency nursing interruption survey.

Index	*X* ^2^/df	RMSEA	IFI	TLI	CFI	PGFI	PNFI
Judgment criteria	<5	<0.08	>0.9	>0.9	>0.9	>0.5	>0.5
Before correction	5.045	0.074	0.908	0.896	0.907	0.704	0.789
After correction	4.661	0.071	0.917	0.906	0.916	0.709	0.794

Abbreviations: CFI, comparative fit index; IFI, incremental fit index; PGFI, Parsimonious goodness of fit index; PNFI, parsimony normed fit index; RMSEA, root mean square error of approximation; TLI, Tucker-Lewis index.

**Table 4:** Convergent validity of the emergency nursing interruption survey questionnaire.

Dimension	Question items	Standardized factor load	CR value	AVE value
Sources	SI1	0.625	0.887	0.570
	SI3	0.745		
	SI4	0.658		
	SI5	0.809		
	SI6	0.848		
	SI7	0.816		

Types	TI1	0.760	0.902	0.699
	TI2	0.824		
	TI3	0.899		
	TI4	0.855		

Nursing procedures	INA1	0.644	0.920	0.593
	INA2	0.788		
	INA3	0.861		
	INA4	0.853		
	INA6	0.768		
	INA7	0.730		
	INA8	0.719		
	INA9	0.775		

Consequences	OINA1	0.567	0.868	0.576
	OINA2	0.580		
	OINA3	0.851		
	OINA4	0.911		
	OINA5	0.818		

Responses	HNIE1	0.717	0.796	0.565
	HNIE2	0.772		
	HNIE3	0.765		

Abbreviations: AVE, average variance extracted; CR, composite reliability; HNIE, handling of nursing interruption events; INA, interrupted nursing activities; OINA, the outcome of interrupted nursing activities; SI, sources of interruption; TI, types of interruption.

## Data Availability

Detailed data are available on request.

## Supporting Information

Supporting Information Appendix 1: Chinese version of Emergency Nursing Interruption Questionnaire.

Supporting Information Appendix 2: English version of Emergency Nursing Interruption Questionnaire.
