# Supplementary material for: Development and Evaluation of Psychometric Properties of a Chinese Version Questionnaire for Measuring Emergency Nursing Interruptions
Source: J Nurs Manag. 2024 Oct 23;2024:8750135. doi: 10.1155/2024/8750135 (PMC11919178; doi:10.1155/2024/8750135)
Supplement: Supporting Information — Appendix 1: Chinese version of Emergency Nursing Interruption Questionnaire. [file 8750135.f1.docx]

**Appendix 1 Chinese version of Emergency Nursing Interruptions Questionnaire**

**一、一般资料**

1.性别

○男 ○女

2.年龄________岁

○18-25 ○26-30 ○31-40 ○41-50 ○>50

3.婚姻状况

○未婚 ○已婚 ○离异或其他

4.文化程度

○中专 ○大专 ○本科 ○硕士及以上

5.职称

○护士 ○护师 ○主管护师 ○副主任护师 ○主任护师

6.急诊护理工作年限（年）

○1-5 ○6-10 ○11-15 ○16-20 ○>20

**二、急诊护理中断事件调查问卷**

**（一）中断的来源**

1.护理工作中断**最多见于工作中的环境与设施设备因素（**如对讲机、办公电话、120 电话、HIS系统、处理打印机或计算机设备故障，制度不完善，工作流程不畅通，突发事件等）。

○非常同意 ○比较同意 ○些许同意 ○比较不同意 ○非常不同意

2.护理工作中断**最多见于护士自身及其护士同事**（如和其他护士的个人电话、沟通交流、身体不适等）。

○非常同意 ○比较同意 ○些许同意 ○比较不同意 ○非常不同意

3.护理工作中断**最多见于患者及家属**（如患者或其家属因为病情、治疗、护理、检查、费用和其他问题影响护士正在进行的工作）。

○非常同意 ○比较同意 ○些许同意 ○比较不同意 ○非常不同意

4.护理工作中断**最多见于医生**（如解决医生的医嘱问题、与医生沟通等）。

○非常同意 ○比较同意 ○些许同意 ○比较不同意 ○非常不同意

5.护理工作中断**最多见于各类学员**（如进修、规培、轮转、实习、专科护士等）。

○非常同意 ○比较同意 ○些许同意 ○比较不同意 ○非常不同意

6.护理工作中断**最多见于其他工作人员**（如营养师、放射技师、后勤支持人员如转运工人、财物人员等）。

○非常同意 ○比较同意 ○些许同意 ○比较不同意 ○非常不同意

**（三）中断的类型**

1.**侵扰型中断（Intrusion）**是最常见的急诊护理中断事件类型*（*侵扰型中断指意外的行为使护理工作变得不连贯）*。

○非常同意 ○比较同意 ○些许同意 ○比较不同意 ○非常不同意

2.**分心型中断（Distraction）**是最常见的急诊护理中断事件类型*（*分心型中断主要是心理上的干扰，当受到外界刺激时，个人不能集中于主要工作）。*

○非常同意 ○比较同意 ○些许同意 ○比较不同意 ○非常不同意

3.**矛盾型中断（Discrepancy）**是最常见的急诊护理中断事件类型（**矛盾型中断指理论或期望的活动与实际工作冲突而产生的中断*）。

○非常同意 ○比较同意 ○些许同意 ○比较不同意 ○非常不同意

4.**毁损型中断（Break）**是最常见的急诊护理中断事件类型（**毁损型中断指工作中有计划或自发性发生的，打断工作连续性，或毁坏主要工作流程的行为*）。

○非常同意 ○比较同意 ○些许同意 ○比较不同意 ○非常不同意

**（四）被中断的护理活动**

1.急诊**分诊活动**最容易被打断。

○非常同意 ○比较同意 ○些许同意 ○比较不同意 ○非常不同意

2.**交接班活动（**如床旁交接班、口头交班、物资交接等）最容易被打断。

○非常同意 ○比较同意 ○些许同意 ○比较不同意 ○非常不同意

3.**基础性护理活动**（如患者的口腔护理、皮肤护理、尿管护理等)最容易被打断。

○非常同意 ○比较同意 ○些许同意 ○比较不同意 ○非常不同意

4.**治疗性护理活动**（如输液、输血、吸痰、采血、振动排痰、吸氧等）最容易被打断。

○非常同意 ○比较同意 ○些许同意 ○比较不同意 ○非常不同意

5.**抢救相关活动**（如抢救病人时的治疗性护理事物、抢救病人时的观察与记录、安置呼吸机、安置心电监护及抢救物资的添加与准备等）最容易被打断。

○非常同意 ○比较同意 ○些许同意 ○比较不同意 ○非常不同意

6.**一般观察与记录性活动**（如书写病程记录、观察病情等）最容易被打断。

○非常同意 ○比较同意 ○些许同意 ○比较不同意 ○非常不同意

7.**健康教育活动**最容易被打断。

○非常同意 ○比较同意 ○些许同意 ○比较不同意 ○非常不同意

8.**上述护理活动之外的其它护理活动**最容易被打断。

○非常同意 ○比较同意 ○些许同意 ○比较不同意 ○非常不同意

**（五）护理活动被中断的结局**

1.急诊护理中断事件的结局**多半是积极的**（如对更重要的工作或任务留

出时间或空间，解决更紧急的事情等）。

○非常同意 ○比较同意 ○些许同意 ○比较不同意 ○非常不同意

2.急诊护理中断事件**对本次护理活动或操作没有影响**。

○非常同意 ○比较同意 ○些许同意 ○比较不同意 ○非常不同意

3.急诊护理中断事件的结局**多半是消极的，以延长某项工作/操作的完成**

**时间为主**。

○非常同意 ○比较同意 ○些许同意 ○比较不同意 ○非常不同意

4.急诊护理中断事件的结局**多半是消极的，以影响护理工作质量为主**。

○非常同意 ○比较同意 ○些许同意 ○比较不同意 ○非常不同意

5.急诊护理中断事件的结局**多半是消极的，以产生护理不良事件为主**。

○非常同意 ○比较同意 ○些许同意 ○比较不同意 ○非常不同意

**（六）对护理中断事件的处理**

1.急诊护理中断事件发生后，您的**首要处理措施多为立即中断（**暂停当前的护理活动，立即处理新发事件）。

○非常同意 ○比较同意 ○些许同意 ○比较不同意 ○非常不同意

2.急诊护理中断事件发生后，您的**首要处理措施多为稍缓中断**（继续当前的护理活动，稍后处理新发事件）。

○非常同意 ○比较同意 ○些许同意 ○比较不同意 ○非常不同意

3.急诊护理中断事件发生后，您的**首要处理措施多为拒绝中断**（继续当前的护理活动，不处理新发事件）。

○非常同意 ○比较同意 ○些许同意 ○比较不同意 ○非常不同意
