# Supplementary material for: Development and Evaluation of Psychometric Properties of a Chinese Version Questionnaire for Measuring Emergency Nursing Interruptions
Source: J Nurs Manag. 2024 Oct 23;2024:8750135. doi: 10.1155/2024/8750135 (PMC11919178; doi:10.1155/2024/8750135)
Supplement: Supporting Information — Appendix 2: English version of Emergency Nursing Interruption Questionnaire. [file 8750135.f2.docx]

**Appendix 2 English version of Emergency Nursing Interruptions Questionnaire**

A. General information

1. Gender

○ Male ○ Female

2. Age (years)

○ 18–25 ○ 26–30 ○ 31–40 ○ 41–50 ○ >50

3. Marital status

○ Unmarried ○ Married ○ Divorced or other

4. Education background

○ Technical secondary school ○ Junior college ○ Undergraduate ○ Master’s degree or above

5. Professional Title

○ Nurse ○ Nurse practitioner ○ Head nurse ○ Associate director nurse ○ Director nurse

6. Experience in emergency nursing (years)

○ 1–5 ○ 6–10 ○ 11–15 ○16–20 ○>20

B. Questionnaire on emergency nursing interruptions

(I) Source of the interruptions

1. The interruption of nursing work is mostly seen in the environment and facilities and equipment factors at work (such as intercom, office telephone, 120 telephone, HIS system, dealing with printer or computer equipment failure, imperfect system, workflow is not smooth, emergencies, etc.).

○ Strongly agree ○ Somewhat agree ○ Somewhat disagree ○ Strongly disagree

2. Interruption of nursing work is most common among nurses themselves and their colleagues (such as personal phone calls, communication with other nurses, physical discomfort, etc.).

○ Strongly agree ○ Somewhat agree ○ Somewhat disagree ○ Strongly disagree

3. Interruption of nursing work occurs most often in patients and their families (if the patient or their family members are affected by the nurse's ongoing work due to illness, treatment, nursing, examination, cost and other issues).

○ Strongly agree ○ Somewhat agree ○ Somewhat disagree ○ Strongly disagree

4. Interruptions in nursing work are most common among doctors (e.g., solving problems with doctors' orders, communicating with doctors, etc.).

○ Strongly agree ○ Somewhat agree ○ Somewhat disagree ○ Strongly disagree

5. Interruption of nursing work is most common in all types of trainees (such as advanced study, training, rotation, internship, specialist nurses, etc.).

○ Strongly agree ○ Somewhat agree ○ Somewhat disagree ○ Strongly disagree

6. Interruptions in care work are most common among other staff (e.g., dietitians, radiographers, logistics support personnel such as transport workers, property personnel, etc.).

○ Strongly agree ○ Somewhat agree ○ Somewhat disagree ○ Strongly disagree

(II) Types of interruptions

1. Intrusion is the most common type of emergency care interruption (*Intrusion refers to unexpected behavior that makes the care work incoherent).

○ Strongly agree ○ Somewhat agree ○ Somewhat disagree ○ Strongly disagree

2. Distractions are the most common type of emergency care interruption (*Distractions are primarily psychological disturbances that prevent individuals from focusing on their main tasks when exposed to external stimuli).

○ Strongly agree ○ Somewhat agree ○ Somewhat disagree ○ Strongly disagree

3. Discrepancy is the most common type of emergency care interruption event (*Discrepancy refers to a conflict between a theoretical or desired activity and actual work).

○ Strongly agree ○ Somewhat agree ○ Somewhat disagree ○ Strongly disagree

4. A disruptive break is the most common type of emergency care interruption (*A disruptive break is a planned or spontaneous occurrence at work that interrupts work continuity or disrupts a major workflow).

○ Strongly agree ○ Somewhat agree ○ Somewhat disagree ○ Strongly disagree

(III) Interrupted nursing activities

1. Emergency triage activities are most likely to be interrupted.

○ Strongly agree ○ Somewhat agree ○ Somewhat disagree ○ Strongly disagree

2. Shift handover activities (such as bedside shift, oral shift, material transfer, etc.) are most likely to be interrupted.

○ Strongly agree ○ Somewhat agree ○ Somewhat disagree ○ Strongly disagree

3. Basic care activities (such as oral care, skin care, urinary tube care, etc.) are most likely to be interrupted.

○ Strongly agree ○ Somewhat agree ○ Somewhat disagree ○ Strongly disagree

4. Therapeutic nursing activities (such as transfusion, blood transfusion, sputum aspiration, blood collection, vibration expectoration, oxygen inhalation, etc.) are most likely to be interrupted.

○ Strongly agree ○ Somewhat agree ○ Somewhat disagree ○ Strongly disagree

5. Rescue related activities (such as therapeutic nursing, observation and record, placement of ventilator, placement of ECG monitoring and addition and preparation of rescue materials, etc.) are most likely to be interrupted.

○ Strongly agree ○ Somewhat agree ○ Somewhat disagree ○ Strongly disagree

6. General observation and recording activities (such as writing disease course records, observing disease conditions, etc.) are most likely to be interrupted.

○ Strongly agree ○ Somewhat agree ○ Somewhat disagree ○ Strongly disagree

7. Health education activities are most likely to be interrupted.

○ Strongly agree ○ Somewhat agree ○ Somewhat disagree ○ Strongly disagree

8. Nursing activities other than those described above are most likely to be interrupted.

○ Strongly agree ○ Somewhat agree ○ Somewhat disagree ○ Strongly disagree

(IV) Consequences of interrupted nursing activities

1. More often than not, the consequences of an emergency nursing interruptions are positive (e.g., for more important work or tasks, make time or space for more urgent things, etc.)

○ Strongly agree ○ Somewhat agree ○ Somewhat disagree ○ Strongly disagree

2. The emergency nursing interruptions has no impact on this nursing activity or operation.

○ Strongly agree ○ Somewhat agree ○ Somewhat disagree ○ Strongly disagree

3. The consequences of an emergency nursing interruptions are likely to be negative, mainly to prolong the completion time of a certain job/operation.

○ Strongly agree ○ Somewhat agree ○ Somewhat disagree ○ Strongly disagree

4. The consequences of emergency nursing interruptions are mostly negative, mainly affecting the quality of nursing work.

○ Strongly agree ○ Somewhat agree ○ Somewhat disagree ○ Strongly disagree

5. The consequences of emergency nursing interruptions are mostly negative, mainly resulting in adverse nursing events.

○ Strongly agree ○ Somewhat agree ○ Somewhat disagree ○ Strongly disagree

(V) Management of nursing interruptions

1. After an emergency nursing interruptions occurs, your first action is usually immediate interruption (suspension of current care activities and immediate treatment of new incidents).

○ Strongly agree ○ Somewhat agree ○ Somewhat disagree ○ Strongly disagree

2. When an emergency nursing interruptions occurs, your first action is usually to slow the interruption (continue with current care activities and deal with new incidents later).

○ Strongly agree ○ Somewhat agree ○ Somewhat disagree ○ Strongly disagree

3. After an emergency nursing interruptions occurs, your first action is usually to refuse the interruption (continue the current care activities, do not deal with new incidents).

○ Strongly agree ○ Somewhat agree ○ Somewhat disagree ○ Strongly disagree
